# Extra-nasal odorant receptors: molecular mechanisms and therapeutic implications

**DOI:** 10.3389/fphys.2026.1869917

**Published:** 2026-09-14

**Authors:** Trung Thach

**Affiliations:** Department of Biological Sciences, Purdue University, West Lafayette, IN, United States

**Keywords:** cryo-EM, drug discovery, extra-nasal, GPCR, odorant receptor

## Abstract

Since the first demonstration of extra-nasal odorant receptor (OR) function of OR1D2 (hOR17-4) in human sperm, ORs have emerged as a widely distributed receptor family. ORs are now known to be belonged to a family of seven transmembrane domain G protein–coupled receptors (GPCRs) expressed across diverse extra-nasal tissues, including skin, gastrointestinal tract, kidneys, cardiovascular system, liver, immune cells, and tumors. In these tissues, ORs act as metabolite- and environment-sensitive sensors that translate chemical cues into tissue-specific signaling pathways that regulate proliferation, differentiation, metabolic homeostasis, and immune responses among others. Dysregulated ORs signaling is increasingly linked to cancer, metabolic, and impaired tissue repair, thereby constitute potential therapeutic targets. Recent advances in cryo-electron microscopy provide near-atomic structural insights into human ORs structure-function, revealing conserved activation mechanisms and ligand-binding features. In parallel, AI-driven molecular dynamics simulation, single-cell transcriptomics, and high-throughput deorphanization platforms are rapidly expanding the OR–ligand landscape. This review integrates current progress in ORs structures and signaling, and outlines key opportunities and challenges for translating extra-nasal ORs structure-function into clinical applications.

## 1 Introduction

The discovery of olfactory receptors as the molecular basis of odor perception by Buck and Axel in 1991 marked a fundamental step in our understanding of sensory biology. It revealed that odor detection is mediated by a large multigene family of seven transmembrane domain G protein–coupled receptors (GPCRs) expressed in olfactory sensory neurons of the nasal epithelium (Buck and Axel, 1991). This landmark discovery, which earned Buck and Axel the 2004 Nobel Prize in Physiology or Medicine, established the receptor family as the primary molecular mediator of chemosensation, and positioned them within the broader class A GPCR superfamily; thereby linking olfactory signaling to conserved principles of GPCR-mediated signal transduction (**Figure 1**). It is worth noting that the term “olfactory receptor” is often used broadly to encompass several distinct chemosensory GPCR families involved in chemical detection, including odorant receptors, vomeronasal receptors, trace amine-associated receptors, and related families, each with distinct expression patterns, ligand classes, and signaling mechanisms (Purves et al., 2001; Fleischer et al., 2009; Jiang and Matsunami, 2015; Korsching, 2025). In this review, we focus specifically on odorant receptors (ORs), the largest and well-characterized of these families, while acknowledging that some of the broader principles discussed may extend to these related receptor classes.

**Figure 1:**
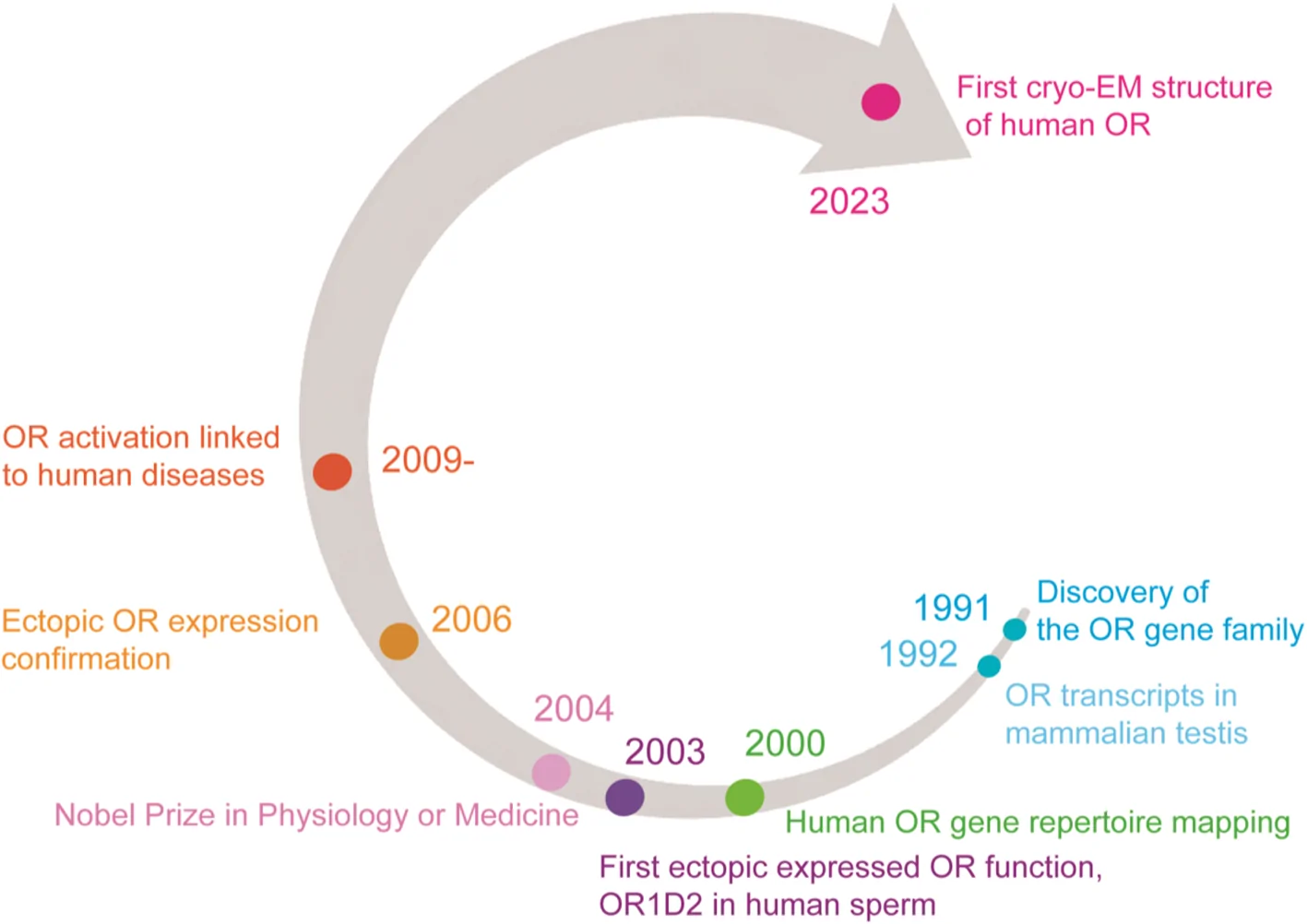
ORs: from sensory discovery to structural biology. The timeline highlights over three decades of progress, beginning with the landmark identification of the OR multigene family as the primary sensors of smell in 1991, and OR transcripts in mammalian testis in 1992. Early research focused on the topographic organization of receptor inputs within the nasal epithelium and the full characterization of the human OR repertoire in the 2000s. A paradigm shift occurred with the discovery of widespread ORs expression in extra-nasal tissues from 2003-2009, leading to the implication of ORs in human pathologies such as prostate cancer, wound healing, and microbiota–host communication. The timeline culminates in the recent resolution of the first atomic-level cryo-EM structure of an OR–G protein complex in 2023.

Genomic analyses revealed that ORs constitute the largest multigene superfamily in the human genome, comprising approximately 400 functional genes and over 600 pseudogenes distributed across nearly all chromosomes (Glusman et al., 2000; Niimura, 2012). This extraordinary genetic expansion reflects strong evolutionary pressure for chemical diversification, enabling organisms to detect a vast spectrum of volatile ligands. ORs are now understood to form combinatorial coding systems in which structurally diverse odorants are recognized by overlapping receptor subsets, thereby generating high-dimensional sensory representations of chemical space (B. Malnic et al., 1999). The degree of OR pseudogenization varies markedly across species. Rodents retain over 1,000 functional OR genes, whereas primates, including humans, exhibit extensive pseudogenization consistent with a reduced reliance on volatile chemosensation, a pattern likely driven by the evolutionary acquisition of trichromatic color vision (Niimura, 2012).

For many years, OR biology was assumed to be restricted to the nasal epithelium. Although Parmentier and colleagues in 1992 reported on OR transcripts in mammalian testis (Parmentier et al., 1992), the field-defining proof of extra-nasal OR function came about a decade later with the landmark study by Spehr and colleagues in 2003. They identified, cloned, and functionally expressed OR1D2 (hOR17-4), a human testicular OR enriched in the midpiece of spermatozoa (Spehr et al., 2003) (**Figure 1**). This study established a proof-of-concept for the entire field. ORs can be coupled to physiologically meaningful downstream signaling pathways, cAMP production, cyclic nucleotide channel opening, and calcium ion influx in extra-nasal cells, which are amenable to pharmacological manipulation by both agonists and antagonists. Critically, chemosensory guidance of gamete navigation toward the oocyte is a conserved reproductive function that predates the nasal system in evolutionary terms (Dubey et al., 2026; Flegel et al., 2013).

Following this proof-of-concept, transcriptomic, proteomic, and functional studies over the subsequent two decades have comprehensively documented widespread ORs expression across extra-nasal tissues. ORs transcripts and proteins have been detected in multiple tissues and organs, including skin, skeletal muscle, lung, gastrointestinal tract, kidney, cardiovascular system, liver, reproductive tissues, and immune cells (Feldmesser et al., 2006; Kang and Koo, 2012; Dubey et al., 2026; Flegel et al., 2013; Maßberg and Hatt, 2018). This unexpected distribution, substantially confirmed by single-cell sequencing and spatial transcriptomic atlases, suggests that ORs represent a broadly deployed chemosensory module rather than a sensory system confined to nasal perception (Glusman et al., 2001; Pourmorady et al., 2024).

Extra-nasal ORs function as metabolite- and environment-responsive GPCRs that couple ligand binding to intracellular signaling networks in a tissue-dependent manner (Maßberg and Hatt, 2018). Depending on the cell type that expresses ORs, its activation engages canonical and non-canonical GPCR signaling pathways, including cAMP production, calcium ion mobilization, mitogen-activated protein kinases (MAPK) activation, and β-arrestin–dependent signaling scaffolds. These enable diverse functional outputs such as secretion, cellular proliferation, differentiation, and immune reaction modulation (Jones and Reed, 1989; Mashukova et al., 2006; Maßberg and Hatt, 2018; Yu et al., 2020). Importantly, this signaling diversity is not intrinsic to the receptor alone but emerges from receptor coupling plasticity shaped by tissue-specific expression of G proteins, including both Gαolf and Gαs, accessory factors, and cell membrane microenvironments.

Functionally, extra-nasal ORs have been implicated in a growing range of physiological and pathological processes (Lee et al., 2019; Yuan et al., 2025: Gu et al., 2025). In epithelial tissues, OR2AT4 activation in keratinocytes promotes wound healing by enhancing cell migration and proliferation in response to synthetic odorants such as Sandalore (Busse et al., 2014; Chéret et al., 2018; Choi et al., 2024). More recently, aptamer-based strategies targeting OR2AT4 have also been explored as a means of promoting hair follicle regeneration, further expanding the therapeutic potential of this receptor (Mataix et al., 2025). In metabolic and gastrointestinal systems, receptors such as OR51E2 respond to short-chain fatty acids and other microbiota-derived metabolites to regulate cellular proliferation and differentiation programs in colon and prostate tissues (Abaffy et al., 2024; Pronin and Slepak, 2021). In the cardiovascular system, OR6A2 expressed in vascular macrophages senses lipid peroxidation products and activates nucleotide binding oligomerization, leucine reach repeats and pyrin domain containing protein 3 (NLRP3)-dependent inflammatory signaling, linking oxidative stress to atherosclerosis progression (Orecchioni et al., 2022b). Collectively, these findings support a model, in which ORs act as distributed chemosensory regulators that integrate endogenous metabolic cues with local tissue function (**Table 1**).

**Table 1:** Extra-nasal ORs implicated in physiology and disease.

OR*	Tissue expression	Key ligands	Cellular function	Disease association	References
*OR51E2* *(Olfr78)*	Prostate, colon, lung epithelium Melanoma; LNCaP cells	β-Ionone, propionate, acetate, Short-chain fatty acids (C2–C9)	- Inhibits proliferation and triggers apoptosis in prostate cancer. - Receptor KO accelerates tumor aggressiveness and STAT3 activation via IL-6. - Drives neuroendocrine trans-differentiation; regulates renin release.	Prostate cancer (suppressor/biomarker), colorectal cancer, asthma, hypertension	(Neuhaus et al., 2009; Weber et al., 2017; Abaffy et al., 2024; Pronin and Slepak, 2021; Billesbølle et al., 2023; Thomsen et al., 2025; Pluznick, 2014; Pluznick et al., 2013)
*OR51E1*	Prostate, heart, Taste cells, intestinal L-cells, Skeletal muscle	Butyrate, valerate, nonanoate (C3–C9 aliphatic acids), Geraniol	- Suppresses prostate cancer cell proliferation. - Stimulates intestinal L-cell GLP-1 secretion to improve	Prostate cancer, Type 2 diabetes (GLP-1 axis), Heart failure, Gustatory disorders	(Han et al., 2018; Pluznick et al., 2013)
*OR51B5*	Acute Myeloid Leukemia (AML) blast cells	Isononyl alcohol	- Activates adenylyl cyclase to elevate cAMP levels. - Induces intracellular Ca^2+^ influx via cyclic nucleotide-gated (CNG) channels to drive myeloblast proliferation.	Acute myeloid leukemia (pro-leukemic driver)	(Manteniotis et al., 2016)
*OR51B4*	Colorectal cancer tissue and HCT116 lines	Troenan (privet flower odorant)	- Inhibits colorectal cancer cell proliferation and migration. - Triggers apoptosis *in vitro* and suppresses metastasis in xenograft models.	Colorectal cancer, (tumor suppressor context)	(Weber et al., 2017)
*OR51V1 (Olfr544)*	Liver hepatocytes, adipose tissue, skeletal myotubes, gut cells	Azelaic acid, Suberic acid	- Mediates PKA-dependent adipocyte lipolysis and accelerates hepatic fatty acid oxidation (FAO). - Shifts systemic fuel preference toward fats; promotes gut GLP-1 secretion.	Obesity, Hepatic steatosis, Intestinal/Colonic inflammation	(Kang et al., 2015; Thach et al., 2017; Wu et al., 2017; Thach et al., 2020)
*OR14I1*	Epithelial cells	HCMV pentameric glycoprotein complex (viral ligand)	- Acts as an obligate cell-surface entry receptor for Human Cytomegalovirus (HCMV). - Hijacks host adenylate cyclase–PKA signaling to drive downstream AKT-mediated endocytosis.	HCMV epithelial infection (therapeutic target)	(E et al., 2019)
*OR10J5 (Olfr16)*	Liver hepatocytes, keratinocytes, olfactory neurons	*α*-Cedrene, lyral-like compounds	- Suppresses hepatic triglyceride accumulation via lipid clearance pathway activation. - Promotes epidermal keratinocyte differentiation.	Hepatic steatosis/NAFLD, Metabolic syndrome	(Tong et al., 2017; Ben Khemis et al., 2022)
*OR6A2 (Olfr2)*	Vascular and aortic macrophages, Bone marrow-derived macrophages	Octanal (lipid peroxidation product of oleic acid), Octanol, medium-chain aldehydes	- Senses volatile lipid peroxidation aldehydes to trigger NLRP3 inflammasome assembly. - Stimulates pro-inflammatory.	Atherosclerosis, Cardiovascular inflammatory disease, Plasma octanal as potential biomarker	(Shi et al., 2024; Orecchioni et al., 2022b; Wang et al., 2026; Qiao et al., 2022)
*OR5V1* *(Olfr110)*	Liver hepatocytes, hypothalamus, testicular tissue	12(S)-HEPE(endogenous oxylipin), PL45(unsaturated fatty acid)	- Acts as a high-affinity endogenous oxylipin sensor activating *G_s_*-PKA-pATF2 signaling. - Enhances hepatic FAO; genomic deletion induces severe glucose intolerance.	Obesity, Insulin resistance, Non-alcoholic fatty liver disease (NAFLD)	(Han et al., 2026; Ge et al., 2026)
*OR5B21*	Metastatic breast cancer lines; bone, lung, brain metastatic niches	unconfirmed	- Enriched in highly metastatic, tissue-specific secondary cancer niches. - Promotes cell invasion and primes cells for epithelial-to-mesenchymal transition (EMT).	Breast cancer distant metastasis (oncogenic driver)	(Li et al., 2021)
*OR4M1 (Olfr734)*	Liver hepatocytes, Hypothalamic neurons, Adipose tissue	Asprosin (adipokine/glucogenic hormone)	- Transduces hepatic gluconeogenic effects of asprosin. - Contributes to anorexigenic signaling within hypothalamic circuits. - Governs the adipose-liver-brain endocrine regulatory axis.	Type 2 diabetes Obesity/appetite dysregulation Hepatic glucose overproduction	(Liu et al., 2020; Li E. et al., 2019; He and Wang, 2022; Xu et al., 2024)
*OR2W3*	smooth muscle cells	Nerol (monoterpene alcohol)	- Induces targeted intracellular Ca^2+^ influx. - Modulates a TMEM16A-/CFTR-dependent chloride current trade-off to promote airway relaxation.	Asthma, COPD (bronchodilation therapeutic target)	(Huang et al., 2020)
*OR2T6*	Breast cancer cell lines Gastric cancer tissue & cell lines	unconfirmed	- Activates downstream MAPK/ERK signaling Cascades. - Drives extracellular Ca^2+^ influx, stabilizes PPP3CA, and promotes EMT phenotypic shifts.	Breast cancer progression, gastric cancer cell proliferation and tumor growth	(Li M. et al., 2019; Yan et al., 2026)
*OR2AT4*	Skin keratinocytes, hair follicle ORS, Leukemia cells (AML/CML)	Sandalore, EGCG, Cyclohexyl salicylate (antagonist)	- Accelerates keratinocyte migration/proliferation to enhance wound closure. - Delays hair follicle regression (prolongs anagen phase). - EGCG sensing induces apoptosis.	Wound healing disorders, androgenetic alopecia, leukemia	(Busse et al., 2014; Chéret et al., 2018; Manteniotis et al., 2016; Choi et al., 2024)
*OR2K2*	Human choroid plexus; brain neurons	unconfirmed	- Distinctly mapped to epithelial cells of the blood-CSF barrier. - Serves as a potential non-invasive sentinel marker for early sporadic neurodegeneration.	Alzheimer’s disease (biomarker), neuromodulation	(Alves et al., 2024).
*OR1D2* *(hOR17-4)*	Spermatozoa (midpiece), mature testes, uterus, prostate	Bourgeonal (agonist), undecanal (competitive antagonist), lyral	- Triggers cAMP-mediated CNG channel opening and subsequent Ca^2+^ influx. - Directs positive sperm chemotaxis and chemokinesis toward the oocyte; SNP leads to motility loss.	Male infertility, sperm chemotaxis failure	(Neuhaus et al., 2006; Spehr et al., 2003; Flegel et al., 2013, 2015)
*OR1A1 (Olfr43)*	Liver hepatocytes (HepG2), Intestinal L-cells, Airway smooth muscle	(−)-Carvone, citral, geraniol Related monoterpenes; lyral-like aldehydes	- Suppresses intracellular lipid accumulation via transcriptional downregulation of PPAR$\gamma$. - Ameliorates hepatic steatosis and improves insulin sensitivity *in vivo*.	Non-alcoholic fatty liver disease (NAFLD), Metabolic syndrome Obesity	(Stary et al., 2007; Kim et al., 2017; Geithe et al., 2017; Wu et al., 2019)

*OR (Human/Mouse Ortholog). 19-OHAD, 19-Hydroxyandrostenedione; AML, Acute Myeloid Leukemia; CML, Chronic Myeloid Leukemia; GLP-1, Glucagon-Like Peptide-1; MAPK, Mitogen-Activated Protein Kinase; NLRP3, NOD-, LRR- and Pyrin Domain-Containing Protein 3 (Inflammasome); HCMV, Human Cytomegalovirus.

Despite their biological and clinical relevance, molecular basic studies of ORs among GPCRs remain challenging. More than 75% of human ORs are still classified as orphan receptors with unknown endogenous ligands, and their functional characterization is hindered by inefficient heterologous expression, intracellular retention, and limited membrane trafficking (Zhuang and Matsunami, 2008; Mainland et al., 2014). Moreover, traditional ligand screening approaches often fail to capture the complexity of tissue-specific signaling environments required for faithful ORs activation, limiting the translational exploitation of this receptor family. Historically, the structural study of ORs was severely hampered by their low expression levels, poor stability, and propensity for intracellular retention.

Recent technological advances are beginning to overcome these barriers. Breakthroughs in cryo-electron microscopy (cryo-EM) have enabled visualization of ORs activation states at near-atomic resolution, providing the first complete structural frameworks for understanding odorant recognition and receptor activation. These include the structure of human OR51E2 in complex with propionate and a miniGαs heterotrimer, the first ectopic OR structure resolved at near-atomic resolution (Billesbølle et al., 2023); the human OR52 consensus receptor (OR52cs) solved in both ligand-free and octanoate-bound active states repertoire (Choi et al., 2023); consensus receptor designs spanning the OR1, OR2, OR4, and OR51 families, providing a broad structural blueprint across the human OR repertoire (de March et al., 2024); the Class II consensus receptor OR6 (OR6cs) bound to α-hexylcinnamaldehyde in complex with mini-Gαolf, revealing a reversible covalent Schiff base mechanism unique among GPCRs (Wang et al., 2026); and the mouse Class II receptor OR5V1/Olfr110 bound to the endogenous oxylipin PL45 in complex with Gαs (Han et al., 2026) (**Table 2**). In parallel, synthetic biology tools, high-throughput functional screening systems, and machine learning–based ligand prediction frameworks are accelerating the systematic deorphanization of ORs. These are enabling structure-guided discovery of receptor modulators.

**Table 2:** Structural insights of representative olfactory receptors.

Receptor	Key structural features	Binding pocket & ligand	Activation mechanism	Key insight
OR51E2 (cryo-EM) PDB: 8F76 EMDB: 28896	- Active (propionate-bound, Gs-coupled) class A GPCR - Compact orthosteric pocket (TM3/5/6/7)	Compact pocket (~31 Å³); short-chain fatty acids (C2–C5), β-ionone	ECL3 closure upon ligand binding; TM6 outward displacement; Na^+^ site stabilizes inactive state	Tight steric filtering defines short-chain ligand selectivity
OR52cs (cryo-EM) PDB: 8W77, 8HTI, 8J46 EMDB: 37336, 35010, 35971	- Engineered consensus (OR52 family) - Dynamic ECL2 lid regulates ligand access	Expanded pocket; medium-chain fatty acids (C5–C10), octanoate (C8)	Dynamic ECL2 lid; ligand-induced ECL–TM6 closure (~7.4 Å); TM6 outward motion	Pocket expansion enables chain-length tuning within OR52 family
consOR1/2/4/51/5 (cryo-EM) PDB: 8UXY, 8UYO, 8UYQ, 8UXV, 9WPM EMDB: 42789–66145	- Consensus designs spanning 17 ORs - Conserved TM3/5/6/7 binding core - Subfamily-specific pocket diversity	Diverse ligand classes: terpenoids (L-menthol), aromatics, SCFAs	Class II activation triad (D^45^·^5^¹–Y^6^·^55^–Y^7^·^4^¹); ligand-dependent conformational selection	Subfamily-specific pocket diversity underlies broad odorant recognition
OR6A2 (cryo-EM) PDB: 9LDV–9LE2 EMDB: 63007–63013	- Class II OR with multiple ligand-bound states - Reversible Schiff base (K157^4^·^60^) - Conserved Class II activation triad	Aldehydes (C8–C10 optimal), aromatic aldehydes; reversible Schiff base (K157^4^·^60^)	Ligand-specific conformations; covalent (reversible) linkage; Class II activation triad	First GPCR with reversible covalent odorant binding mechanism
Olfr110 (cryo-EM) PDB: 9LKB, 9LKD EMDB: 63174, 63175	- Class II OR; human-OR5V1 ortholog - Shared Class II activation triad - Greater flexibility vs. Class I ORs	Very large pocket (~1500 Å³); long-chain lipids, 12(S)-HEPE, PL45	TM6 displacement at both ends (~4.1 Å); flexible binding cavity; Gs/Golf coupling	Adapted for sensing bulky lipid metabolites with expanded pocket architecture

consOR, consensus olfactory receptor; PDB, Protein Data Bank; SCFA, short-chain fatty acid; 12(S)-HEPE, 12(S)-hydroxyeicosapentaenoic acid; ECL, extracellular loop; TM, transmembrane helix.

This review summarizes current advances in our understanding of human ORs and their well-studied murine orthologs beyond the nasal system, with an emphasis on their structural basis, tissue distribution, and context-dependent signaling mechanisms. We then discuss their emerging roles in key physiological and pathological processes, including metabolism, cancer, immune regulation, and tissue regeneration. Finally, we highlight major conceptual and technical challenges in the field and outline future directions for translating extra-nasal ORs biology into therapeutic applications.

## 2 Molecular basis of extra-nasal odorant receptor function

ORs are class A (rhodopsin-like) GPCRs characterized by a conserved seven transmembrane domain (7TM) architecture but extreme sequence diversification (Niimura, 2012; Qin et al., 2025). Each OR gene is typically intronless that encodes approximate 310–330 amino acid-protein comprising seven transmembrane helices, an extracellular N-terminus, and an intracellular C-terminal tail. Functional specificity arises primarily from hypervariable residues within TM3–TM6, which form the ligand-binding cavity that exhibit strong signatures of adaptive evolution, enabling broad ligand affinities while maintaining conserved downstream signaling pathways (Man et al., 2004; Glusman et al., 2001).

### 2.1 Classification and evolutionary divergence of odorant receptors

To contextualize the structural and functional diversity of ORs discussed throughout this review, we conducted a comprehensive phylogenetic analysis using human OR sequences alongside their corresponding murine orthologs retrieved from the NCBI database. Maximum-likelihood analysis of the aligned sequences produced a radial tree in which ORs resolve into two well-supported, reciprocally monophyletic clades corresponding to Class I and Class II (**Figure 2**), consistent with the deep evolutionary split previously documented (Niimura, 2012).

**Figure 2:**
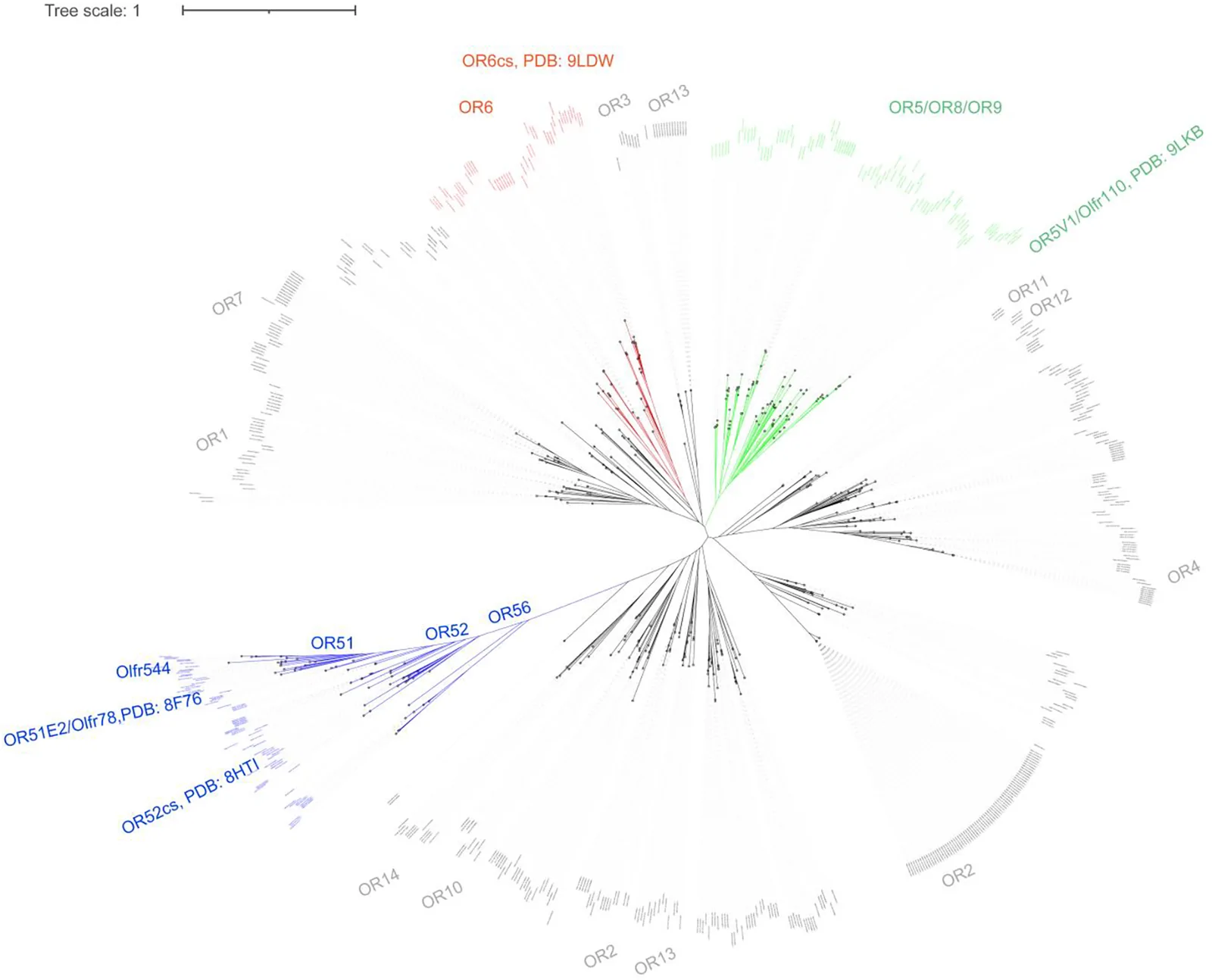
Phylogenetic analysis of human ORs and their well-characterized murine orthologs. Target OR protein sequences were programmatically retrieved from the NCBI RefSeq database and subjected to multiple sequence alignment using MAFFT v7.526 via the progressive FFT-NS-2 heuristic strategy (Katoh and Standley, 2013). To remove poorly aligned, highly divergent, and gap-rich positions common to transmembrane GPCR loop regions, the raw alignment was automatically trimmed using TrimAl v1.4 (Capella-Gutiérrez et al., 2009). Phylogenetic tree reconstruction was performed via maximum likelihood using IQ-TREE 3 (Wong et al., 2026), utilizing ModelFinder (Kalyaanamoorthy et al., 2017) for rapid candidate substitution model selection (-m TEST) and 1,000 ultrafast bootstrap replicates to calculate node branch support (Hoang et al., 2018).The resulting Newick tree topology was formatted and annotated for final interactive visualization using iTOL v5 (Letunic and Bork, 2021). The python scripts and source code are available upon request.

Class I ORs form a compact, clearly delineated clade (blue, **Figure 2**) comprising the three human subfamilies OR51, OR52, and OR56, with approximately 55 functional members. Despite representing only ~14% of the functional human OR repertoire by gene number, Class I occupies a phylogenetically coherent and well-supported branch, reflecting its origin as a conserved ancestral lineage present in aquatic vertebrates prior to the tetrapod radiation (Niimura, 2012; Yohe et al., 2020). Functionally, Class I ORs are tuned to detect relatively hydrophilic ligands, most notably short-chain aliphatic carboxylic acids and water-soluble volatile compounds, consistent with a chemosensory system ancestrally adapted to detect chemicals dissolved in an aquatic medium (Zhang and Firestein, 2002). This ligand preference is directly supported by the cryo-EM structures resolved for two Class I receptors: OR51E2 (PDB: 8F76) binds propionate, a three-carbon carboxylate (Billesbølle et al., 2023); and the OR52 consensus receptor (OR52cs, PDB: 8HTI) binds octanoate, an eight-carbon carboxylate (Choi et al., 2023). Within our phylogenetic tree, these structural models map precisely to their expected lineages: human OR51E2 and its murine ortholog Olfr78 cluster; OR51V1 and its closely related murine counterpart Olfr544 within the OR51 subfamily, while OR52cs segregates within the OR52 subfamily (Bulger et al., 2000) (**Figure 2**). This structural-to-phylogenetic alignment validates the accuracy of our tree topology.

Class II ORs form the large, expansive clade (gray, green and red, **Figure 2**), encompassing subfamilies OR1 through OR14 and approximately 336 functional genes, roughly 86% of the functional human OR repertoire. The Class II clade is substantially larger and more internally diverse than Class I, with fourteen families that can be grouped into higher-order phylogenetic clusters reflecting shared evolutionary origins rather than fourteen fully independent lineages (Niimura, 2012; Yohe et al., 2020) (**Figure 2**). The expansion of Class II ORs reflects adaptation to complex volatile environments, driving a functional shift toward larger, more hydrophobic compounds including aldehydes, ketones, esters, and aromatic molecules (Zhang and Firestein, 2002). Several receptors central to this review are located within the Class II clade: OR1A1 and OR1D2 within the OR1 subfamily; OR2AT4, OR2J3, and OR2W3 within the OR2 family, the largest and most diversified Class II subfamily; and OR6A2 within the phylogenetically more isolated OR6 family, which is the basis of one of the four cryo-EM structures discussed here (PDB: 9LDW) (red, **Figure 2**). Notably, Olfr110 (human ortholog OR5V1) (PDB: 9LKB) clusters within the Class II OR5/OR8/OR9 clade (green, **Figure 2**), phylogenetically distinct from OR6, which illustrates a key point: the phylogenetic distance between different Class II families is substantial (Niimura, 2012; Ge et al., 2026).

### 2.2 Comparative analysis of OR ligand-binding pockets

OR ligand binding occurs within a deeply buried orthosteric pocket formed primarily by the transmembrane bundle core of TM3, TM5, TM6, and TM7, with contributions from extracellular loop 2 (ECL2) (**Figure 3**). Unlike classical GPCRs that often possess rigid, highly tailored binding cavities, ORs display expanded and conformationally flexible ligand-binding pockets. The structural plasticity allows receptors to accommodate chemically diverse volatiles and metabolites, ranging from short-chain fatty acids to bulky aromatic compounds. This pocket plasticity is beautifully illuminated by the four currently resolved OR–ligand complexes: OR51E2 bound to propionate (PDB: 8F76), OR52cs bound to octanoic acid (PDB: 8HTI), OR6cs bound to α-hexylcinnamaldehyde (PDB: 9LDW), and OR5V1/Olfr110 bound to the oxylipin metabolite PL45 (PDB: 9LKB). Rather than relying on a rigid template, these structural snapshots demonstrate how a flexible binding architecture undergoes precise local configurations tailored to the specific chemical class of its cognate ligand (**Figures 3A–C**; **Table 2**).

**Figure 3:**
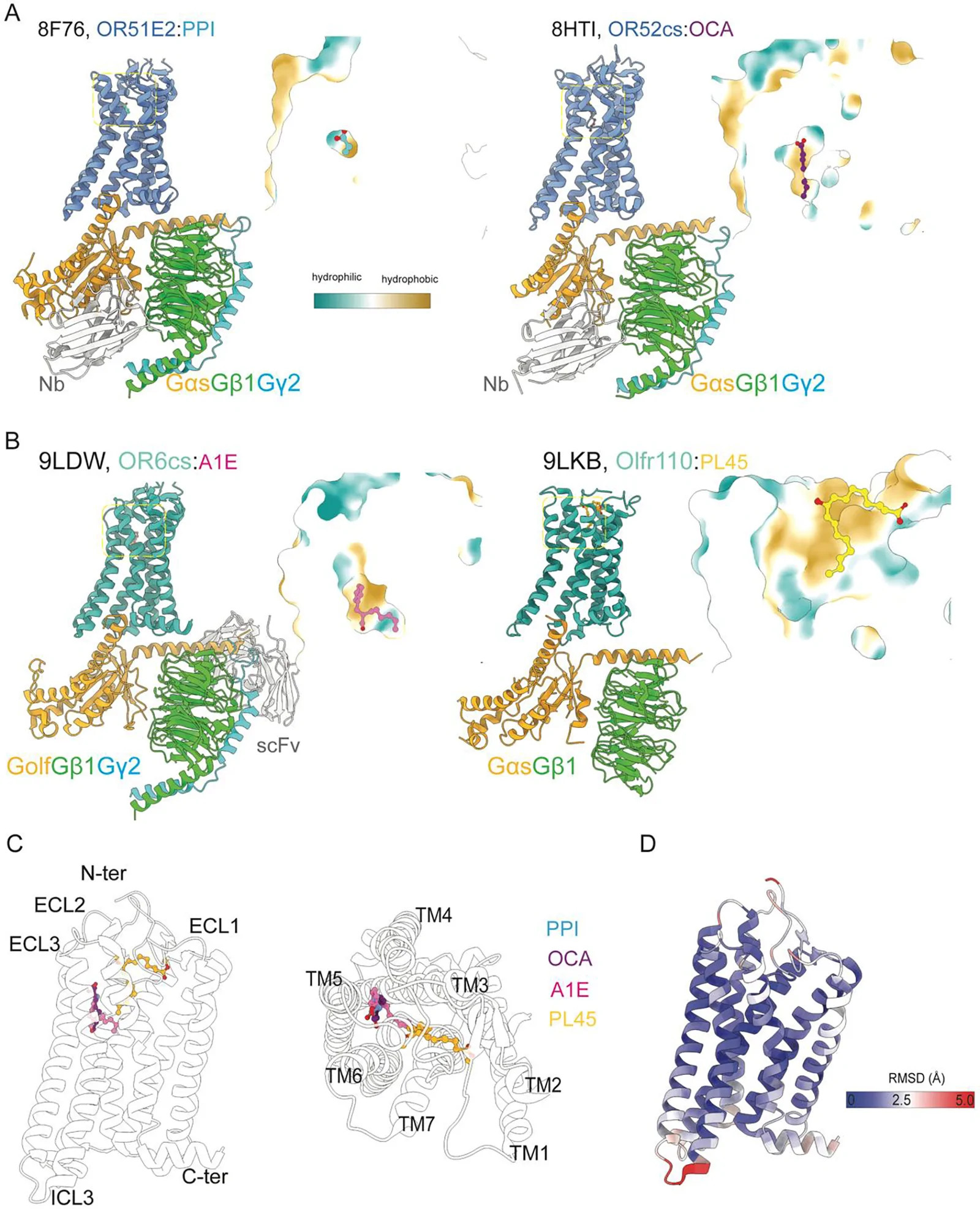
Structural basis of odorant receptor-ligand recognition. **(A, B)** Cryo-EM structures of ligand-bound OR–G protein complexes. Global views of ligand-bound, signaling-active complexes representing Class I **(A)** and Class II **(B)**. Four resolved OR structures are shown: Class I receptor complexes of OR51E2–propionate–miniGαs (PDB: 8F76) and OR52cs–octanoate–Gαs (PDB: 8HTI); Class II consOR6–α-hexylcinnamaldehyde–miniGαolf (PDB: 9LDW) and Olfr110–PL45–Gαs (PDB: 9LKB). Insets: Magnified cross-sections of the orthosteric binding pockets overlaid with hydrophobicity surface mapping (teal: hydrophilic; white: neutral; gold: hydrophobic). Receptor chains are colored by blue; Gα subunits in green, Gβ in orange, Gγ in cyan. Ligands shown as stick-ball. Nb, nanobody, scFv, single-chain fragment variable antibody. **(C)** Ligand-binding pocket: Comparative side and top-down views showcasing pocket topology variations. Class I binding cavities (OR51E2, OR52cs) are compact and primarily coordinated by residues along TM3, TM5, and TM6 to accommodate short-chain, water-soluble ligands. Conversely, the Class II OR6cs pocket exhibits a larger, bipartite geometry, while Olfr110 possesses the most expanded binding cavity to accommodate its bulky, unsaturated fatty acid metabolite PL45. **(D)** Structural comparison of the four target OR chains demonstrating strict fold conservation, yielding a RMSD of 1.1–1.6 Å over 299 equivalent C*α* atoms. Significant backbone divergence is localized almost exclusively to the highly flexible ECL2 and ICL3 loops. All structural models, hydrophobicity analysis, and RMSD calculations were generated using UCSF ChimeraX.

In OR51E2 (PDB: 8F76), the ligand-binding cavity is exceptionally small and deeply occluded, yielding a restrictive pocket volume for short-chain carboxylates. It accommodates a three-carbon carboxylate, propionate (PPI, MW ~74 Da), through tight steric packing with surrounding TM4, TM5, and TM6 residues. This physical constraint excludes ligands longer than approximately five carbons, demonstrating that selectivity in Class I ORs is governed primarily by steric complementarity (Billesbølle et al., 2023) (**Figures 3A, C**; **Table 2**). In OR52cs (PDB: 8HTI), representing a distinct sub-lineage within Class I, the orthosteric site of receptor is slightly wider and deeper than OR51E2 (**Figure 3A**). It incorporates a specialized, elongated hydrophobic channel line by TM5 and TM6 residues (**Figures 3A, C**). This unique channel accommodates the linear eight-carbon aliphatic tail of octanoic acid (OCA, MW ~144 Da), explaining the receptor’s preference for C6–C10 chain-length recognition while maintaining a tightly sealed extracellular loop (Choi et al., 2023) (**Figures 3A, C**; **Table 2**).

In OR6cs (PDB: 9LDW), the orthosteric pocket of receptor exbibits a volumetric expansion compared to its Class I couterparts (**Figures 3A, B**). It features a wide bipartite architecture that binds to the reactive aromatic aldehyde head group and the six-carbon aliphatic tail of α-hexylcinnamaldehyde (A1E, MW ~230 Da) through a combination of aromatic sub-pocket contacts and hydrophobic channel (Wang et al., 2026). Strikingly, Olfr110 (PDB: 9LKB) possesses an unusually large hydrophobic binding pocket, the most expanded orthosteric cavity structurally characterized in ORs to date. The pocket transitions from a deep TM core anchor to an expansive, solvent-exposed extracellular vestibule (**Figure 3B**). This massive volume is capable of accommodating the bulky, elongated 18-carbon PL45 (methyl (9R,10E,12E)-9-methoxyoctadeca-10,12-dienoate (MW ~340 Da). The pocket is decorated by polar residues engaging the C9-methoxy group and aromatic residue arrays forming π–π interactions with the conjugated diene at C10–C12, recognition features entirely absent from the purely aliphatic pockets of OR51E2 and OR52 (Han et al., 2026) (**Figures 3A–C**; **Table 2**). This progressive expansion of pocket volume, propionate (C3) → octanoate (C8) → α-hexylcinnamaldehyde (C15, aromatic) → PL45 (C18, methoxy-diene), directly confirms that Class I ORs generally accommodate smaller, more hydrophilic ligands while Class II ORs favor larger, more lipophilic volatile and metabolite-derived compounds.

Importantly, direct structural comparison highlights the core Cα backbones of these four OR receptors aligning with a root-mean-square deviation (RMSD) of 1.1–1.6 Å over 299 equivalent atoms (**Figure 3D**). This statistical proximity proves that the dramatic variations in pocket volume and geometry are not caused by massive shifts in the overall protein fold. Instead, this geometric divergence is driven locally by the distinct rotameric packaging of inward-facing side chains along TM3, TM5, and TM6, alongside the highly variable conformation of the ECL2 loop ceiling, providing an elegant structural explanation for how the uniform GPCR scaffold was evolutionarily adapted to perceive an infinite universe of chemical shapes.

### 2.3 Three mechanistically distinct ligand recognition strategies

Across the four representative structures, the mode of polar recognition at the ligand functional group diverges fundamentally. In both OR51E2 (PDB: 8F76) and OR52cs (PDB: 8HTI), the anionic carboxylate of the fatty acid ligand forms a non-covalent ionic interaction with a conserved arginine at Ballesteros–Weinstein position 6.59 in TM6. In OR52cs, R^6^·^59^ is the primary anchor, and its mutation abolishes the cAMP response to octanoate in concentration–response assays, confirming its functional necessity (Choi et al., 2023). This arginine–carboxylate salt bridge is fully reversible and concentration-dependent, representing fundamental GPCR pharmacology. In OR6cs (PDB: 9LDW), a distinctly different strategy operates: the aldehyde carbonyl of α-hexylcinnamaldehyde reacts nucleophilically with the ϵ-amino group of a conserved lysine at position K^4^·^60^ in TM4, forming a reversible covalent Schiff base imine (C=N bond) (Wang et al., 2026). This is unique among all characterized GPCRs. The phenyl ring of α-hexylcinnamaldehyde simultaneously occupies an aromatic sub-pocket, providing a dual-anchor recognition element, covalent head group attachment combined with aromatic pocket engagement, that is inaccessible to the purely aliphatic carboxylate ligands of OR51E2 and OR52cs. In Olfr110 (PDB: 9LKB/9LKD), recognition is mediated by a third, distinct strategy: the methoxy group at C9 of PL45 participates in a polar interaction network formed by pocket-lining polar residues, while the conjugated diene at C10–C12 engages aromatic residues through π-stacking contacts, a mode of recognition that requires neither a charged head group nor a reactive carbonyl. Instead, it relies on the specific geometry of oxygenated and unsaturated functional groups distributed along a long-chain lipid scaffold (Han et al., 2026). The structural coexistence of these three recognition chemistries, non-covalent ionic (8F76, 8HTI), reversible covalent Schiff base (9LDW), and polar network plus π-stacking (9LKB/9LKD), within the same conserved 7-TM fold establishes that OR pharmacology cannot be reduced to a single pharmacophoric model, and that drug or fragrance design developments targeting different OR families would be tailored to the specific recognition chemistry of each target.

### 2.4 Extracellular gating: ECL3 remodeling, TM5–TM6 gap closure, and bilateral TM6 displacement

Cryo-EM structures of ligand-bound ORs indicate that odorant binding induces localized conformational rearrangements in extracellular loops, particularly ECL2 and ECL3. These act as dynamic gating elements regulating ligand entry and pocket stabilization (**Figure 3**, **Table 2**). In OR51E2-propionate complex, molecular dynamics simulations demonstrate that propionate-induced conformational changes in ECL3 are the primary extracellular activation trigger: ECL3 remodels from a disordered conformation to a compact, closed structure that caps the binding pocket and initiates the allosteric signal propagating toward the TM core (Billesbølle et al., 2023). In OR52cs receptor, a mechanism was revealed through comparison with the companion apo structure (PDB: 8W77): the pre-existing 14 Å gap between TM5 and TM6 visible in the ligand-free state functions as the actual ligand entry route, which closes inward by 7.4 Å at the extracellular face of TM6 upon octanoate binding. The intracellular TM6 end simultaneously moves outward to open the G protein cavity in active state (Choi et al., 2023). This dual-direction “crank” motion of TM6 is mechanistically distinct from the ECL3-dominated gating of OR51E2, and provides a direct structural explanation for how the blocking of the TM5–TM6 cleft with an allosteric modulator antagonizes OR52 family members through a non-competitive mechanism.

In Olfr110 receptor, the inward movement of ECL3 is again the primary extracellular event upon PL45 binding. Interestingly, the associated TM6 displacement occurs bilaterally, with both the extracellular end (Cα of 6.56, ~ 4.1 Å outward) and the intracellular end moving outward, a simultaneously outward bilateral TM6 displacement not observed in Class I OR structures (Han et al., 2026). In OR6cs, the reversible covalent Schiff base chemistry at K^4^·^60^ introduces a kinetic dimension absent from the other three systems. The covalent intermediate stabilizes the receptor–ligand complex beyond the residence time achievable by non-covalent contacts alone. This potentially sustains activation in tissues such as macrophages where receptor expression is lower than in primary sensory neurons, and where even moderate increases in receptor occupancy could translate into significantly amplified downstream NLRP3 inflammasome signaling (Wang et al., 2026).

### 2.5 Conserved intracellular activation and G protein coupling

Despite ligand-specific differences in the extracellular orthosteric pocket, the downstream intracellular activation mechanism remains highly conserved across all four structures. Agonist binding within the core cavity induces a concerted outward displacement of the cytoplasmic end of TM6, creating the intracellular cavity required for insertion of the Gs α5-helix (**Figure 4**). This helical transition is coordinated by fundamental class A GPCR microswitches. Specifically, activation drives a spatial rearrangement of the PIF toggle across the TM5-TM6 interface, a conformational shift in the conserved DRY (Asp-Arg-Tyr) motif in TM3, and a corresponding rotational inward movement of the NPxxY motif in TM7 (**Figure 4**). Consistent with classical class A GPCR activation paradigms, this transmission network involves the structural disruption of the TM3–TM6 ionic lock, a prominent inward pivot of TM7, and a complete packing reorganization across the TM5–TM6 helical interface.

**Figure 4:**
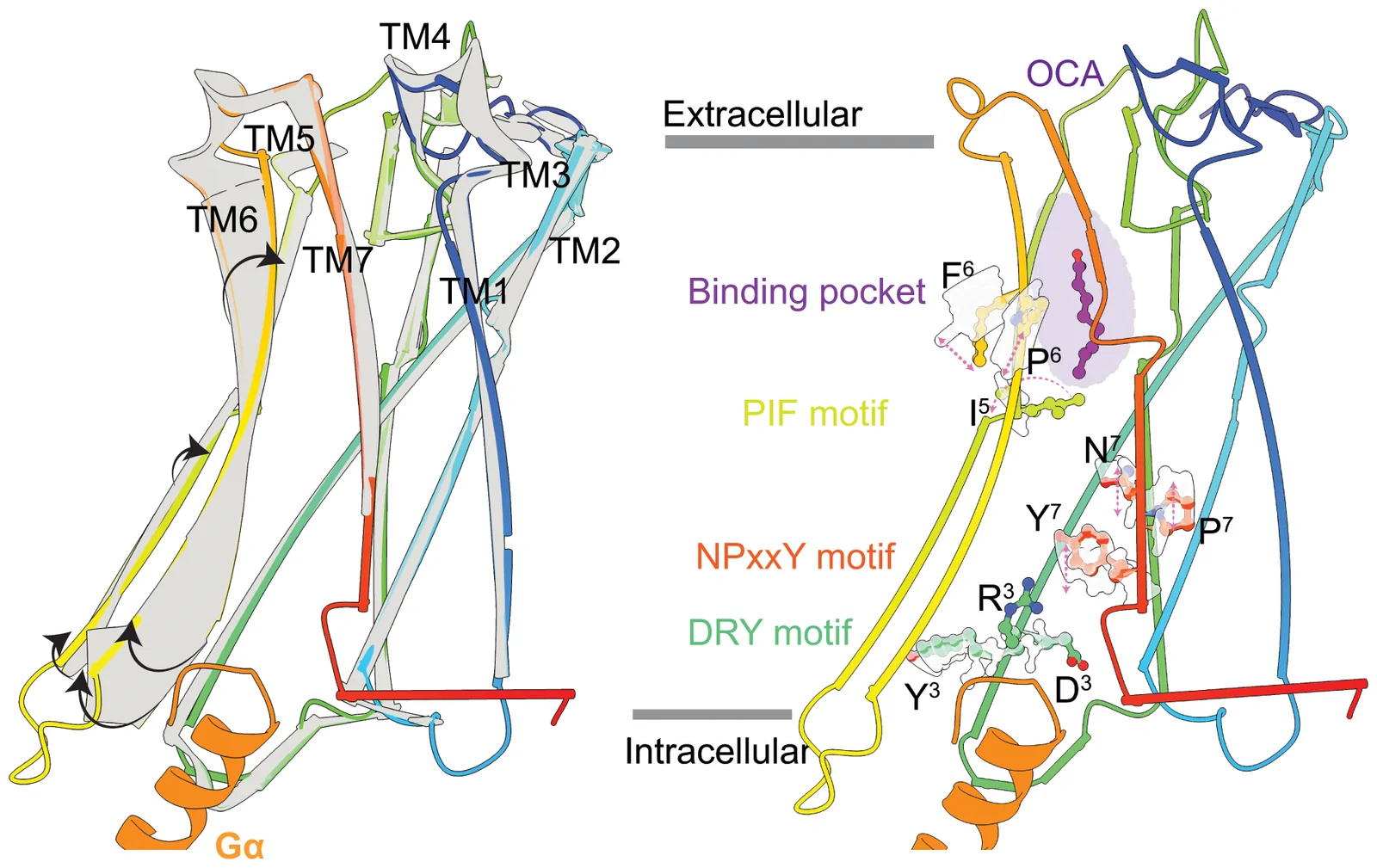
Molecular basis of ligand-induced OR activation. Structural superposition of apo and odorant-bound active conformations of OR52cs (PDB: 9W77 and 8HTI). The morph trajectory, generated in ChimeraX, is shown as grey cartoon tube helices, with the active state rendered as rainbow one. Ligand binding induces conformational rearrangements that disrupt the conserved TM3–TM6 ionic lock and reposition key microswitch motifs, including the PIF toggle (TM5–TM6), the DRY motif (TM3), and the NPxxY motif (TM7). These transitions collectively create a cytoplasmic cavity that accommodates the Gα α5-helix (orange). Black arrows indicate the outward displacement of TM6, associated movement of TM5, and conserved motifs during activation. Structure figures were generated using UCSF ChimeraX.

Notably, ORs exhibit greater structural and conformational plasticity than most classical GPCR families, suggesting a more flexible allosteric landscape underlying receptor state transitions. Within this shared structural framework, however, a profound class-level divergence emerges within the intracellular allosteric signaling network. Comparative structural analysis of Class I structures (OR51E2, OR52cs) and Class II structures (OR6cs, Olfr110) reveals distinct activation anchors: Class I ORs fundamentally rely on a network stabilized by the highly conserved residues D^5^·^50^ (82% conservation) and R^4^·^5^² (88%). Conversely, Class II ORs bypass this network, utilizing a D/E^45^·^5^¹–Y^6^·^55^–Y^7^·^4^¹ triad, validated in OR6cs (9LDW) and applicable to Olfr110. This triad is essentially absent in Class I receptors. Strikingly, the primary Class I anchor, D^5^·^50^, exhibits a conservation profile of mere ~0.3% in Class II lineage, highlighting a fundamentally distinct intracellular signaling architecture and diverging evolutionary solutions for G-protein coupling (Wang et al., 2026; Han et al., 2026) (**Figure 4**). This class-specific intracellular fingerprint has direct, critical implications for structure-based drug discovery and functional annotation. Because of these distinct active-state topologies, structural homology models, virtual ligand screening campaigns, and allosteric modulator design strategies derived from Class I templates like OR51E2 are highly likely to misrepresent or fail when applied to the intracellular signaling pharmacology of Class II targets (such as OR6A2 or OR5V1/Olfr110), and vice versa.

### 2.6 Intracellular diversity in extra-nasal ORs’ signaling

Each olfactory sensory neuron (OSN) expresses a single OR gene from the large functional receptor repertoire, a phenomenon known as the “one neuron–one receptor” rule (Chess et al., 1994; Serizawa et al., 2003; Mombaerts, 2004). This monogenic and monoallelic expression is governed by a stochastic, irreversible gene-choice mechanism driven by multi-locus enhancer competition, feedback suppression from the successfully translated OR protein (Lomvardas et al., 2006; Magklara et al., 2011). Additional studies suggest that interactions between distant regulatory DNA elements (enhancers) further stabilize this process, helping ensure that each OSN maintains expression of a single OR gene throughout its lifetime (Sharma et al., 2017).

At the systems level, these individually specialized OSNs implement a combinatorial coding strategy, in which odorants are recognized by overlapping subsets of broadly tuned receptors (Malnic et al., 1999). Consequently, odor identity and intensity are represented as a high-dimensional population code across the nasal epithelium rather than a simple labeled-line circuit. Recent structural biology breakthroughs provide a clear mechanistic basis for this cross-reactive tuning. Variations in orthosteric pocket volume and the local hydrophobic environment shape ligand selectivity across different receptor families (Billesbølle et al., 2023; Choi et al., 2023; Han et al., 2026; Wang et al., 2026).

Importantly, in extra-nasal tissues, the canonical one-neuron-one-receptor rule established in OSN has not been demonstrated, as non-neuronal cells are not known to implement the specialized OR gene-choice mechanism. Multiple ORs have been reported to be expressed within the same extra-nasal tissues (Flegel et al., 2013; Maßberg and Hatt, 2018), although direct evidence for their co-expression in individual cells remains limited. Consequently, the combinatorial logic governing odor coding in the olfactory epithelium may not directly translate to extra-nasal OR signaling. If multiple ORs are co-expressed within the same cell, they could potentially compete for shared G proteins, converge on common downstream effectors, or generate additive or antagonistic signaling outputs depending on ligand availability and receptor expression levels.

A key implication is that receptor function is not intrinsically “nasal” or “extra-nasal” but is cell-type and tissue dependent. The same binding pocket can support distinct physiological roles depending on the given tissue, ligand availability, and downstream signaling pathway. For example, OR6A2 detects volatile medium-chain aldehydes such as octanal in olfactory sensory neurons, contributing to odor perception. However, in vascular macrophages, the same receptor recognizes octanal coupling detection to NLRP3 inflammasome activation and IL-1β secretion, thereby promoting atherosclerosis (Orecchioni et al., 2022b). Similarly, OR5V1/Olfr110 responds to long-chain odorants in the nasal epithelium, but detects the EPA-derived eicosanoid 12(S)-HEPE in liver and intestine, where it regulates fatty acid oxidation via a Gs–PKA–Cpt1α axis (Han et al., 2026). In both cases, receptor identity is constant; functional output is dictated by the cell type.

In OSNs, ORs couple to Gαolf (encoded by *GNAL*), which activates adenylyl cyclase III (ACIII) to generate cAMP. cAMP opens cyclic nucleotide–gated (CNG) channels, allowing Na^+^ and Ca²^+^ influx and membrane depolarization. The Ca²^+^ increase further amplifies the response via TMEM16B (anoctamin-2, a Ca²^+^-activated Cl^-^ channel) that drives Cl^-^ efflux (Saraiva et al., 2019; Lee et al., 2019). This cascade confers high sensitivity (pM–nM range). Gαolf is functionally distinct from Gαs: it preferentially couples to ACIII, exhibits faster nucleotide exchange, and is largely restricted to OSNs and select neurons. In extra-nasal contexts, if present, it instead engages Adenylyl Cyclase V and/or VI (ACV/VI), altering cAMP dynamics (**Figure 5**).

**Figure 5:**
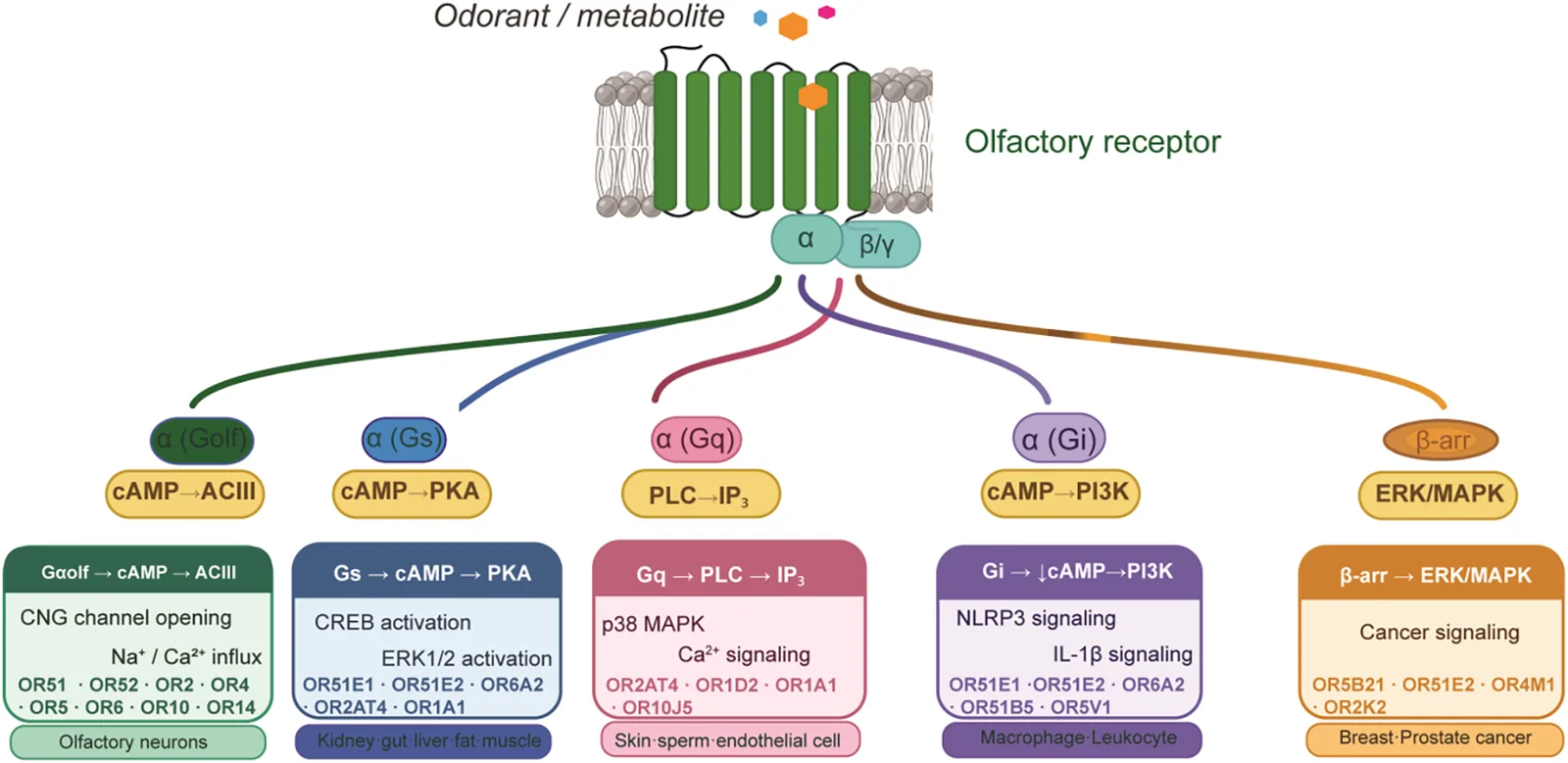
Signaling pathways engaged by ORs in extra-nasal tissues. In olfactory sensory neurons, ORs couple predominantly to Gαolf, which activate adenylyl cyclase III, thereby elevating cAMP and opening cyclic nucleotide–gated (CNG) channels. In contrast, in extra-nasal tissues, ORs couple to multiple signaling mediators, including Gαs, Gαq/11, Gαi/o, and β-arrestins, resulting in diversified downstream outputs. The relative engagement of these pathways varies across cell types, reflecting differences in G protein expression, effector availability, and cellular context, thereby producing tissue-specific physiological outcomes.

Outside the nasal epithelium, ORs couple promiscuously to Gαs, Gαi/o, and Gαq/11, with coupling determined by cellular G protein availability and membrane rather than receptor sequence alone (Lee et al., 2019). Gαs–cAMP–PKA is the predominant pathway: activation of ACV/VI elevates cAMP, driving PKA–CREB signaling. This underlies diverse functions, including induced proliferation and migration in keratinocytes (OR2AT4), metabolic regulation in adipose/liver (Olfr544), and differentiation in epithelial cancers (OR51E2). Structural data for OR6A2 demonstrate dual coupling to Gαolf and Gαs, providing direct evidence that a single receptor can engage different stimulatory G proteins depending on tissue type (Wang et al., 2026). Gαq/11–PLC–IP_3_–Ca²^+^ links OR activation to intracellular Ca²^+^ release and PKC/MAPK signaling. This pathway operates in epithelial cells and the reproductive system, often in combination with cAMP signaling to produce synergistic responses (e.g., OR2AT4 in keratinocytes, OR1D2 in sperm) (Chéret et al., 2018). Gαi/o signaling suppresses cAMP while activating PI3K pathways via Gβγ (Corey et al., 2021). Several ORs, including OR6A2 in macrophages, OR51B5 in Acute Myeloid Leukemia, exhibit dual second messenger responses (cAMP↑ and Ca²^+^↑) (Chung et al., 2022; Orecchioni et al., 2022b; Weidinger et al., 2021; Manteniotis et al., 2016), consistent with concurrent engagement of stimulatory and inhibitory G protein pathways, although direct Gαi coupling remains to be fully established at the structural level (**Figure 5**, **Table 1**).

Upon sustained activation, ORs are phosphorylated by G protein-coupled receptor kinases 2/3 (GRK2/3) at intracellular loop and C-terminal residues, promoting recruitment of β-arrestin-1/2. β-arrestin binding sterically blocks further G protein coupling and facilitates receptor internalization via clathrin-mediated endocytosis. In parallel, Ca²^+^–calmodulin feedback enhances GRK activity and dampens upstream signaling, contributing to tight temporal control of receptor responses (Mashukova et al., 2006). Beyond desensitization, β-arrestins function as signaling scaffolds that assemble MAPK modules, including ERK1/2, p38, and C-Jun N-terminal kinases (JNK), enabling G protein–independent signaling and establishing a biased signaling framework. For example, OR51E2 activation induces sustained, spatially restricted ERK1/2 signaling via a Gβγ–PI3Kγ–ARF1 pathway, consistent with compartmentalized MAPK signaling (Lefkowitz and Shenoy, 2005; Peterson and Luttrell, 2017). Similarly, OR5B21 has been linked to ERK/MAPK-driven proliferation and migration in experimental cancer models, although direct β-arrestin coupling remains to be established (Li et al., 2021). Together, these findings suggest that tissue-specific expression of GRKs and β-arrestins shapes both signal termination and the balance between G protein–dependent and –independent OR outputs (**Figure 5**, **Table 1**).

### 2.7 Signal termination and regulatory control

Termination of ORs signaling involves layered negative-feedback mechanisms that operate at distinct temporal scales. Intracellular cAMP is rapidly hydrolyzed by phosphodiesterases, particularly PDE1C and PDE4 isoforms, restoring basal signaling levels and limiting cyclic nucleotide accumulation (Yan et al., 1995; Lefkowitz and Shenoy, 2005). In parallel, GPCR kinase–mediated phosphorylation of activated ORs promotes β-arrestin recruitment, receptor uncoupling, and clathrin-dependent internalization, thereby attenuating further G protein signaling (Lefkowitz and Shenoy, 2005; Peterson and Luttrell, 2017). In the cell membrane, Ca²^+^–calmodulin provides a rapid, local feedback mechanism by directly inhibiting CNG channels through binding to the CNGB1b subunit, shortening response duration independently of receptor desensitization (Song et al., 2008; Reisert and Zhao, 2011). These mechanisms ensure high temporal precision, prevent saturation, and preserve dynamic range across broad odorant ligands concentrations.

In extra-nasal tissues, analogous termination pathways are engaged but exhibit distinct kinetics due to cell-type-specific expression of phosphodiesterases, GRKs, and Ca²^+^ regulatory proteins. Reduced or altered PDE1C/PDE4 expression can prolong OR-driven cAMP signals, leading to sustained PKA activity and extended transcriptional responses compared with the rapid transients observed in olfactory sensory neurons (Lefkowitz and Shenoy, 2005). This variation in signal termination dynamics is consistent to the divergent physiological outcomes of OR activation across tissues, ranging from transient secretory responses to long-lasting changes in gene expression and cell state.

## 3 Tissue distribution and physiological roles of extra-nasal ORs

Extra-nasal ORs are now recognized as a widely distributed class of chemosensory GPCRs that extend far beyond their fundamental role in odor detection. Although typically expressed at lower levels than in olfactory sensory neurons, these receptors exhibit highly tissue-specific patterns and are often dynamically regulated under physiological and pathological conditions (Feldmesser et al., 2006; Flegel et al., 2013; Lee et al., 2019; Liu et al., 2025). Integration of these expression data with spatial transcriptomics is now enabling the construction of tissue-level ORs distribution maps that link receptor expression to local metabolite environments and disease-associated metabolic states (**Table 1**).

### 3.1 ORs in cancer biology and tumor microenvironment regulation

Aberrant expression of extra-nasal ORs has emerged as a recurrent feature across a broad spectrum of malignancies, including prostate, colorectal, breast, melanoma, and lung cancers. ORs expression profiles are evidenced as tumor-specific, highlighting their potential diagnostic tool as biomarkers (Chung et al., 2022; Neuhaus et al., 2009; Weber et al., 2017; Tang et al., 2025; Thomsen et al., 2025). Rather than acting as classical oncogenes, ORs appear to function as modulators of tumor cell state, influencing proliferation, differentiation, and metabolic adaptation in context-dependent manners (**Figures 5**, **6**; **Table 1**).

**Figure 6:**
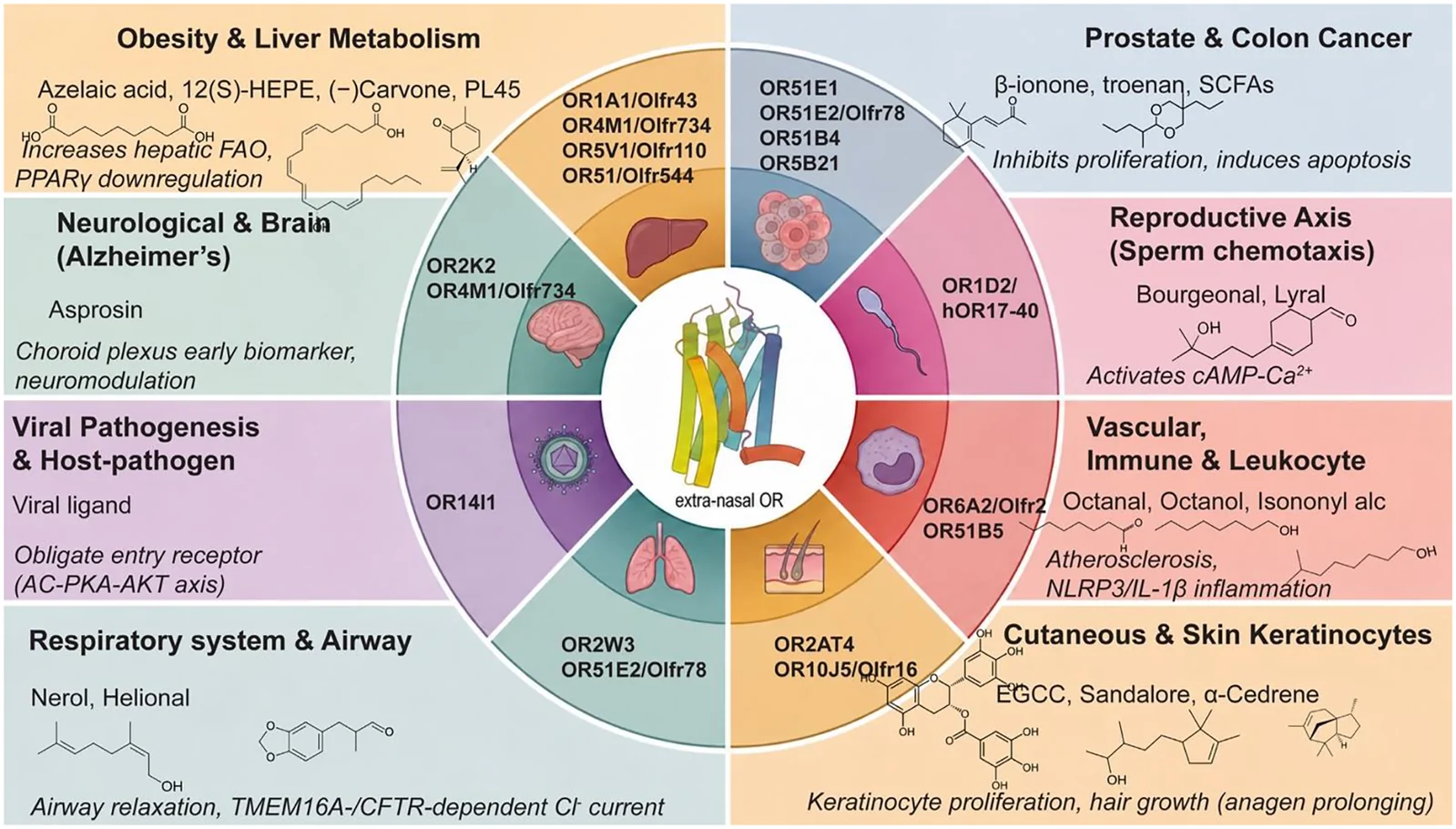
Physiological and pathological roles of representative extra-nasal ORs. Beyond their canonical function in odor detection, ORs are broadly expressed in peripheral tissues, where they act as chemosensory GPCRs responding to endogenous metabolites and environmental ligands. ORs activation regulates diverse cellular processes, including oncogenesis, reproductive axis function, immune regulation, cutaneous physiology and wound healing, respiratory homeostasis, host-pathogen interactions, neurological function and Alzheimer’s disease pathology, and metabolic homeostasis and obesity. These context-dependent functions underscore the dual physiological and pathological roles of extra-nasal ORs.

A well-characterized example is OR51E2 (prostate-specific G protein–coupled receptor, PSGR), which is highly expressed in prostate cancer. Activation of OR51E2 by ligands such as β-ionone and short-chain fatty acids (SCFAs) induces cAMP-dependent signaling that regulates proliferation and promotes differentiation. This also modulates androgen-responsive transcriptional networks (Weber et al., 2017). OR51E2 also functions as a critical homeostatic brake against high-grade tumor progression. Complete genomic knockout of OR51E2 in both *in vitro* cultures and *in vivo* mouse xenograft models triggers a paradoxically hyper-aggressive phenotype, significantly accelerating tumor cell proliferation, anchorage-independent colony formation, and metastatic tissue adhesion (Thomsen et al., 2025). This regulatory axis is strongly corroborated by clinical dataset analyses showing that low tumor expression of OR51E2 tightly correlates with advanced Gleason scores and poor patient survival outcomes (Thomsen et al., 2025). Consequently, the functional outcome of OR51E2 signaling is highly dependent on cellular state, receptor expression levels, and the localized ligand environment, with evidence supporting both protective anti-proliferative roles and complex involvement in metabolic reprogramming (**Figure 6**, **Table 1**).

In colorectal cancer, OR51B4 is upregulated and activated by the synthetic odorant troenan, triggering phospholipase C–mediated Ca²^+^ signaling that reduces cell migration, induces apoptosis, and inhibits tumor growth in xenograft models (Weber et al., 2017). OR2T6 has been linked to breast cancer progression through activation of the MAPK/ERK pathway and induction of epithelial-to-mesenchymal transition (EMT), highlighting the specific pro-tumorigenic potential of some ORs (Li M. et al., 2019). Conversely, a recent study demonstrated that OR2T6 serves a protective, tumor-suppressive function in gastric cancer (Yan et al., 2026). In gastric malignancies, OR2T6 expression is downregulated and correlates with poor patient prognosis; when active, it drives a canonical Gs/cAMP/PKA signaling axis that promotes intracellular calcium (Ca^2+^) influx (Yan et al., 2026). Crucially expanding this breast cancer landscape, OR5B21 has been identified as an oncogenic driver of aggressive distant metastasis. It is significantly enriched in metastatic cell lines targeting the bone, lungs, and particularly the brain, where its overexpression enhances cell invasion and primes cells for EMT (Li et al., 2021).

Beyond solid tumors, OR signaling plays a regulatory role in hematological malignancies, as exemplified by OR2AT4. In human myelogenous leukemia cells, OR2AT4 activation serves as a critical regulator of hematopoiesis and leukemogenesis. Its modulation actively restricts uncontrolled cell proliferation, induces intrinsic apoptosis, and drives myeloid differentiation toward a more mature phenotype (Manteniotis et al., 2016). Highlighting its therapeutic tractability, epigallocatechin gallate (EGCG) was recently identified as a potent natural agonist of OR2AT4, mimicking these anti-proliferative and pro-apoptotic effects in leukemia models and further broadening the scope of OR-centered cancer pharmacology (Choi et al., 2024) (**Figure 6**, **Table 1**).

Beyond these tumor-intrinsic properties, ORs are active participants in tumor microenvironment regulation. Malignant niches are characterized by altered metabolic landscapes, including hypoxia, enhanced glycolysis, and the accumulation of microbial or host-derived volatile organic compounds. Metabolite-sensing ORs on both tumor and stromal cells may act as sensors linking these localized metabolic cues to aggressive tumor phenotypes. Comprehensive evaluations of odor-based therapeutics targeting ORs in cancer underscore the potential for developing OR-directed small-molecule agonists or antagonists that act as effective adjuncts to conventional anti-cancer treatments (Liu et al., 2025).

### 3.2 ORs in metabolic, gastrointestinal, and cardiovascular regulation

Growing volume of evidence indicates that extra-nasal ORs play an important role in systemic metabolic regulation by acting as sensors of nutrient-derived and microbiota-derived metabolites (Pluznick et al., 2013; Ren et al., 2024). Their expression in metabolically active tissues, including the gastrointestinal tract, liver, pancreas, adipose tissue, and skeletal muscle, positions them as key components of inter-organ communication networks governing energy homeostasis (**Figure 5**, **6**).

In the gastrointestinal tract, ORs expressed in enteroendocrine cells respond to SCFAs produced by gut microbiota. OR51E1 is activated by butyrate and related SCFAs and promotes secretion of glucagon-like peptide-1 (GLP-1) from intestinal L-cells, thereby influencing insulin secretion and peripheral glucose metabolism (Han et al., 2018; Pluznick et al., 2013) (**Table 1**). This establishes ORs as part of a broader class of GPCR-based nutrient sensors that link microbial metabolism to host endocrine function. Emerging data further suggest that ORs-mediated sensing of branched-chain amino acids and other microbiota metabolites may contribute to the gut–brain axis, with potential implications for appetite regulation and metabolic disease (Yang et al., 2023).

In peripheral metabolic tissues, a directly functional ortholog of human OR51V1, murine Olfr544 (Bulger et al., 2000; Malnic et al., 2004) is activated by dicarboxylic acids including azelaic acid, inducing lipolysis, fatty acid oxidation, and CREB-dependent transcriptional programs through PKA signaling, thereby promoting energy utilization and reducing adiposity (Kang et al., 2015; Thach et al., 2017; Wu et al., 2017; Thach et al., 2020). OR4M1 (murine ortholog Olfr734) has been identified as a receptor for asprosin, a glucogenic hormone secreted by adipose tissue, providing a direct link between ORs-mediated sensing and glucose metabolism (Liu et al., 2020; Li E. et al., 2019). In the liver, OR10J5 responding to α-cedrene regulates hepatic steatosis through cAMP–PKA signaling (Ben Khemis et al., 2022; Tong et al., 2017), and OR1A1 (murine ortholog Olfr43) activation suppresses lipogenic gene expression by repressing peroxisome proliferator-activated receptor gamma (PPARγ) through HES-1 induction (Stary et al., 2007; Wu et al., 2019; Geithe et al., 2017). Together, these findings indicate that ORs constitute a distributed metabolite-sensing network in metabolic tissues, acting in parallel with classical metabolic hormone receptors.

ORs also contribute to cardiovascular and renal homeostasis. The murine receptor Olfr78, phylogenetically related to human OR51E2, is expressed in renal juxtaglomerular cells and vascular smooth muscle, where it senses circulating SCFAs to regulate renin secretion and systemic vascular tone (Pluznick, 2014; Pluznick et al., 2013). More recently, an evolutionarily conserved OR4M1 has been shown to mediate sex differences in blood pressure regulation, suggesting that OR-mediated signaling may contribute to the known sexual dimorphism in cardiovascular risk (He and Wang, 2022; Xu et al., 2024). In vascular biology, OR6A2 expressed in macrophages binds octanal, a product of lipid peroxidation, and activates NLRP3 inflammasome–dependent IL-1β production, mechanistically linking oxidized lipid sensing to atherosclerosis progression (Shi et al., 2024). These findings identify the OR–NLRP3–IL-1β axis as a potential therapeutic target in cardiovascular inflammatory disease.

### 3.3 ORs in neurological, reproductive, and immune systems

The presence of ORs in the central nervous system suggests that they function beyond classical chemo-sensation. ORs transcripts have been identified in mature neurons, astrocytes, and neural progenitor cells. This raises the possibility that these receptors participate in neuromodulatory processes or metabolic sensing within the brain (Jiang and Matsunami, 2015). Genetic studies have linked polymorphic variation in ORs gene clusters to neuropsychiatric conditions, including susceptibility to schizophrenia, major depressive disorder, and autism spectrum disorders (Trimmer et al., 2019). Furthermore, OR2K2 expression has been mapped to the human choroid plexus, and proposed as a potential non-invasive early biomarker for sporadic Alzheimer’s disease, pointing to a role for OR in neurodegenerative disease surveillance (Alves et al., 2024) (**Figure 6**, **Table 1**).

In the reproductive system, ORs are expressed in spermatozoa, mature testes, uterus, and prostate, where they contribute to sperm chemotaxis, fertilization, and epithelial homeostasis (Neuhaus et al., 2006; Flegel et al., 2013, 2015) (**Figure 6**, **Table 1**). This receptor-mediated chemotactic signaling is vital for sperm navigation through the female reproductive tract toward the oocyte, a physiological journey critically dependent on intracellular cAMP flux and calcium mobilization downstream of OR activation, highlighting an evolutionarily conserved role for ORs in mammalian reproduction (Flegel et al., 2013, 2015). Dysregulation of these same lineages, such as OR51E2, further bridges extra-nasal OR signaling with hormone-responsive tissue pathophysiology and oncogenesis.

Emerging evidence also implicates ORs in immune regulation. ORs expression has been detected across circulating leukocytes and tissue-resident immune cells, where receptor activation modulates cytokine production and innate inflammatory signaling pathways (He and Wang, 2022; Orecchioni et al., 2022b). The mechanistic link between human OR6A2 (murine ortholog Olfr2), octanal sensing, and NLRP3-dependent IL-1β production in macrophages represents one of the most rigorously characterized examples of chemosensory-immune crosstalk, with direct pathological relevance to chronic vascular disease (Orecchioni et al., 2022b; Qiao et al., 2022). Within the microenvironment of an inflamed arterial wall, the accumulation and peroxidation of low-density lipoproteins generate elevated levels of volatile aldehydes, including octanal, which serves as an endogenous danger signal (Wang and Andreasson, 2022). Vascular macrophages expressing OR6A2 detect this lipid peroxidation byproduct, triggering intracellular calcium influx and reactive oxygen species production that functions as a secondary signal to assemble the NLRP3 inflammasome (Qiao et al., 2022). This activation drives the robust secretion of pro-inflammatory cytokines like IL-1β and IL-1α, directly fueling the vascular inflammation that accelerates atherosclerotic plaque progression (Orecchioni et al., 2022a; Wang and Andreasson, 2022) (**Figures 5**, **6**; **Table 1**). Beyond this localized atherogenic axis, broader screenings of leukocyte transcriptomes suggest additional roles for ORs in modulating monocyte and neutrophil migration in response to modified metabolites, as well as altering T-cell activation profiles, though the functional characterization of these immune OR networks remains an active area of investigation (Orecchioni et al., 2022b).

### 3.4 ORs in viral infection and host-pathogen interactions

Beyond systemic homeostatic surveillance, recent studies demonstrate that extra-nasal ORs are active cellular components at the host-pathogen interface, functioning either as direct entry conduits for viral pathogens or as indirect casualties of local viral-induced inflammation.

A premier example of direct exploitation is the human OR14I1, which serves as an essential cell-surface entry receptor defining the epithelial cell tropism of Human Cytomegalovirus (HCMV) (E et al., 2019) (**Figures 5**, **6**; **Table 1**). Genome-wide CRISPR/Cas9 screens have revealed that the multipass membrane architecture of OR14I1 directly binds the highly neutralizing HCMV pentameric glycoprotein complex (E et al., 2019). Rather than operating as a passive tether, HCMV binding triggers the intrinsic signaling capacity of OR14I1, hijacking its canonical adenylate cyclase and protein kinase A (PKA) pathway to drive downstream AKT activation. This virus-induced signaling cascade actively remodels the host cell membrane, facilitating endocytosis-mediated entry and infection of clinically critical epithelial barrier sheets. Targeting this specific chemosensory node using synthetic OR14I1 N-terminal decoy peptides or pharmacological PKA inhibitors potently aborts epithelial infection, positioning OR14I1 as a major therapeutic target for preventing inter-host transmission (E et al., 2019).

Conversely, in the respiratory tract, ORs expressed in bronchial epithelial cells influence inflammatory signaling and responses to pulmonary insults, including viral infections. Notably, transient SARS-CoV-2 infection of non-neuronal sustentacular cells in the olfactory epithelium triggers a profound, non-cell-autonomous disruption of the nuclear architecture within mature OSNs (Zazhytska et al., 2022). Although OSNs lack the ACE2 receptor and are not productively infected by the virus, the localized inflammatory response induces a widespread collapse of the three-dimensional genomic organization within the OSN nuclei (Khan et al., 2021; Zazhytska et al., 2022). Specifically, SARS-CoV-2 infection leads to a severe loss of the interchromosomal genomic compartments and multi-locus enhancer aggregations, structural “hubs” that are absolutely required to drive monogenic OR transcription (Monahan et al., 2019; Zazhytska et al., 2022). This reorganization causes a drastic and sustained downregulation of the entire OR gene expression repertoire and its essential signaling components including adenylate cyclase 3 (Adcy3) and the odorant G-protein alpha subunit (Gnal). This profound loss of primary sensory transcripts offers a precise molecular rationale for why olfactory dysfunction can persist long after active viral clearance (Finlay et al., 2022). This structural vulnerability highlights how local, tissue-specific extra-nasal inflammation can crosstalk with neuronal nuclear homeostasis, reinforcing the paradigm of sensory OR disruption as a highly sensitive sentinel marker of systemic viral disease.

### 3.5 Potential therapeutic opportunities and challenges

The widespread distribution and ligand accessibility of extra-nasal ORs make them attractive, albeit underexplored, candidates for pharmacological targeting. However, the relationship between OR biology and drug discovery requires careful framing, since the broad therapeutic promise of GPCRs as a receptor class does not currently extend to ORs in clinical practice. GPCRs constitute the largest family of drug targets in clinical use, mediating the actions of approximately 36% of all FDA-approved drugs (Hauser et al., 2017; Zhang et al., 2024; Lorente et al., 2025). Of the roughly 800 GPCRs encoded in the human genome, about half are ORs (Hauser et al., 2017; Vedel et al., 2020). Yet almost no OR-targeted therapies have entered clinical development.

This gap arises from three compounding factors. First, the majority of human ORs remain orphan receptors with no confirmed endogenous ligand, representing the single largest unexplored receptor category for ligand-based drug discovery (Jabeen and Ranganathan, 2019). Second, even where ligands are known, they frequently display low binding affinity, often in the micromolar range, and limited receptor selectivity, properties that fall short of the potency and specificity profiles typically required for drug candidates (Berwal et al., 2025; Wetzel et al., 1999; Yuan et al., 2019). Third, most functional and pharmacological data for ORs derive from heterologous expression systems, primarily HEK293 or Hana3A cells co-transfected with accessory chaperones such as RTP1S; these systems are themselves limited by inefficient receptor folding, intracellular retention, and poor plasma membrane trafficking. It remains uncertain how faithfully the resulting pharmacological profiles translate to the native signaling environment of extra-nasal tissues, where G protein availability, accessory proteins, and membrane composition differ substantially from the heterologous (Saito et al., 2004; Yu et al., 2017; Ieki et al., 2022).

Several proof-of-concept studies illustrate where OR-directed pharmacology may eventually translate into therapeutic application, while underscoring that these remain preclinical or early-stage findings rather than established drug targets (**Figure 6**, **Table 1**). In the skin, activation of OR2AT4 by the synthetic sandalwood odorant Sandalore promotes keratinocyte proliferation and migration, accelerating wound healing in ex vivo human skin models (Busse et al., 2014). Aptamer-based OR2AT4 targeting has also been validated as a hair growth–promoting strategy in preclinical models, extending the potential dermatological application of OR2AT4 modulation (Mataix et al., 2025). In oncology, OR51B4 activation by troenan inhibits colorectal tumor cell growth and promotes apoptosis in xenograft models (Weber et al., 2017), while EGCG-mediated OR2AT4 activation exerts anti-leukemic effects in cell-based assays (Choi et al., 2024). In metabolic tissues, OR10J5 and OR1A1 activation modulates hepatic lipid metabolism in rodent models, and OR51E1 activation in enteroendocrine cells enhances GLP-1 secretion *in vitro*, with potential relevance to type 2 diabetes management pending *in vivo* validation (Kim et al., 2017; Tong et al., 2017; Han et al., 2018). In cardiovascular disease, OR6A2 antagonism is being explored at the preclinical stage as a strategy to limit macrophage-driven NLRP3 inflammasome activation and atherosclerosis progression in mouse models (Orecchioni et al., 2022b).

None of these findings have yet progressed to clinical trials. Beyond the orphan-receptor, affinity, and heterologous-system limitations described above, two further obstacles complicate translation. OR signaling is highly cell-dependent: ligand bias and cell-type-specific G protein coupling, as discussed for Gαolf, Gαs, Gαi, and Gαq pathways in earlier sections. This means that a compound’s pharmacological profile measured in a heterologous assay may not predict its functional outcome in the target extra-nasal tissue, complicating both efficacy prediction and safety assessment (Zhuang and Matsunami, 2008; Odoemelam et al., 2025). In addition, the high sequence conservation among OR subfamily members, particularly within the TM3–TM6 ligand-binding core, complicates the development of subtype-selective pharmacological tools, raising concerns about off-target engagement of ORs in the nasal epithelium or of related OR paralogs in other tissues.

A further challenge specific to OR-directed drug discovery concerns the reliability of the expression data used to nominate candidate receptors for a given tissue or disease context in the first place. The transcriptomic and proteomic evidence underlying most extra-nasal OR target hypotheses carries technical caveats that directly affect target validation (Schultz and Coelingh Bennink, 2022; Lalis et al., 2023). Droplet-based single-cell RNA sequencing is susceptible to ambient mRNA contamination, in which cell-free transcripts released during tissue dissociation are captured within unrelated cell-containing droplets. These generate spurious low-level expression signals unless corrected computationally (Yang et al., 2020; Arora et al., 2025). OR transcripts are also frequently expressed at very low copy numbers in extra-nasal tissues, making them particularly vulnerable to dropout events in single-cell sequencing. This issues can cause a genuinely expressed candidate target to be missed entirely, or conversely cause an apparently tissue-restricted target to appear more broadly expressed than it truly is (Yang et al., 2020; Arora et al., 2025). The extensive sequence homology among OR family members, a consequence of the tandem gene duplication events that generated this multigene family, creates substantial mapping ambiguity, as short sequencing reads may align equally well to multiple paralogous OR genes (Hughes et al., 2018; Paz et al., 2024). A particular concern for selectivity assessment, since a candidate nominally directed at one OR paralog may need to be evaluated against several closely related family members whose tissue distribution cannot be (Deschamps-Francoeur et al., 2020). Most critically for translational prioritization, validation at the protein level remains a major bottleneck: the same high sequence similarity that complicates selectivity also makes it exceptionally difficult to generate antibodies with adequate specificity. Transcript-level evidence frequently cannot be confirmed by immunohistochemistry or western blotting in the relevant human tissue, leaving open the question of whether the receptor is present at a level sufficient for pharmacological intervention to have any effect (Kalra et al., 2021; Kahn et al., 2024). Addressing these challenges will require integrated molecular, computational, both *in vitro* and *in vivo* approaches, as described in the following section.

## 4 Recent advances in OR research and future perspectives

### 4.1 Integrated structural biology and AI-driven systems modeling

Recent advances in structural biology and artificial intelligence (AI) are jointly transforming ORs research from a receptor-by-receptor experimental field into a systems-level, predictive discipline. Experimental cryo-EM structures combining with AI-based structure modeling and MD simulation provide complementary, experimentally validated frameworks (Thach et al., 2026). The growing library of ORs cryo-EM structures, spanning OR51E2, OR52 consensus, OR1/2/4/51 consensus families, OR6 consensus, and OR5V1/Olfr110, now offers comprehensive coverage of both Class I and Class II human and mouse ORs (Billesbølle et al., 2023; Choi et al., 2023; de March et al., 2024; Wang et al., 2026; Han et al., 2026). These structures define the full conformational trajectory of OR activation, including the critical TM6 outward displacement, formation of the cytoplasmic G protein cavity, and the distinct agonist-dependent binding modes that govern signaling bias. When integrated with molecular dynamics (MD) simulations, these structures evolve into dynamic conformational ensembles that capture ligand-dependent activation pathways, intermediate states, and allosteric communication networks within the receptor core.

Beyond individual receptor modeling, systems-level approaches integrate ORs structural predictions with transcriptomic, proteomic, and metabolomic datasets. Single-cell RNA sequencing and spatial transcriptomics have revealed highly tissue-specific ORs expression patterns across metabolic, epithelial, immune, and neuronal compartments, enabling mapping of receptor distribution onto functional biological networks (Glusman et al., 2001). Machine learning approaches now extend these capabilities by directly predicting receptor–ligand interactions using hybrid feature sets combining sequence embeddings, structural descriptors, evolutionary coupling signals, and known GPCR–ligand interaction databases (Odoemelam et al., 2025). Importantly, high-resolution pocket metrics derived from these experimental structures are leveraged to train Geometric Deep Learning and Graph Neural Networks capable of mapping 3D binding site topography (Gainza et al., 2020; Townshend et al., 2022; Stephenson and Karnati, 2025). By feeding these structural descriptors into end-to-end AI deorphanization platforms for generative molecular docking, and specialized chemosensory pipelines, researchers can screen massive chemical spaces in silico (Jabeen and Ranganathan, 2019; Odoemelam et al., 2025; Lee et al., 2023). Furthermore, integrating these models with repository frameworks like GPCRdb, GPCRmd allows for the correlation of structural pocket mutations with ligand-binding affinity across entire odorant subfamilies (Herrera et al., 2024; Rodríguez-Espigares et al., 2020). These models can prioritize candidate ligands for orphan ORs, identify chemically convergent receptor subfamilies, and predict tissue-specific signaling outputs with increasing accuracy. These shift OR research from empirical screening toward hypothesis-driven, structure-guided AI-driven deorphanization pipelines.

Emerging modalities such as cryo-focused ion beam milling coupled with cryo-electron tomography and *in situ* structural proteomics further extend this integrative framework into native cellular architectures of OR complexes. This potentially resolves higher-order receptor organization, lipid raft localization, and receptor clustering in intact cells. Together, these advances establish a unified computational–structural–systems biology framework capale of tracking OR function seamlessly from atomic-level chemosensory ligand coordination to tissue-level physiologcial networks.

### 4.2 High-throughput deorphanization and functional screening platforms

A central bottleneck in OR research remains the large number of orphan receptors lacking validated ligands, currently estimated to exceed 75% of functional human ORs. To address this, substantial progress has been made in improving heterologous expression systems and functional screening technologies. Engineered cell platforms such as HEK293 and Hana3A cells co-expressing accessory proteins (RTP1S, RTP2, REEP1, Ric8b, and Gαolf) have significantly enhanced ORs trafficking efficiency and membrane localization. These enable robust functional readouts via cAMP-responsive reporters, calcium imaging, or luciferase-based biosensors (Zhuang and Matsunami, 2008).

High-throughput odorant screening has enabled systematic mapping of receptor–ligand interactions across chemically diverse libraries. This reveals both broad ligand promiscuity and receptor-specific tuning profiles. The compilation of human ORs responses to odorants in publicly available datasets has provided foundational reference points for the field. Databases such as the Molecule to Olfactory Receptor database [M2OR; (Lalis et al., 2023)], the Human Olfactory Data Explorer [HORDE; (Marenco et al., 2016)], and comprehensive multi-species systems like the Chordata Olfactory Receptor Database [CORD; (Han et al., 2025)] have aggregated decades of disparate functional bioassays into standardized, open-access repositories.

Multiplexed deorphanization strategies combining barcoded ORs expression libraries with next-generation sequencing and single-cell transcriptomics have enabled parallel interrogation of hundreds of receptors in a single experimental framework (Lazar and Yeh, 2020; McLaughlin et al., 2021). Bioinformatics platforms such as GPCRdb, updated in 2025 to include odorant receptors, structure similarity search tools, and models of physiological ligand complexes, are now providing integrated structural–functional databases to support systematic ORs annotation (Herrera et al., 2025).

### 4.3 Synthetic biology and engineered ORs systems

Synthetic biology is expanding the functional utility of ORs beyond native chemo-sensation. Rational mutagenesis, directed evolution, and consensus receptor engineering approaches have been used to improve receptor folding efficiency, membrane expression, and ligand sensitivity in heterologous systems (de March et al., 2024; Zhuang and Matsunami, 2008). Chimeric receptor engineering, where ORs extracellular domains are fused to intracellular signaling modules from well-characterized GPCRs, has further improved signal robustness and assay reliability, decoupling ligand recognition from native signaling complexity. These enable quantitative pharmacological analysis of ORs activation (Schulz et al., 2022).

Beyond basic research, ORs-based synthetic circuits are being developed as programmable biosensors. These systems couple ligand detection to gene expression outputs, enabling detection of volatile compounds, metabolic biomarkers, or environmental toxins. The use of ORs as chemical sensing scaffolds in engineered cell systems and cell-free platforms is an active area of development, with proof-of-concept demonstrations in biosensing of medically relevant volatile organic compounds (Patel and Peralta-Yahya, 2023; Zhang et al., 2022). Such engineered platforms highlight the translational potential of ORs in diagnostics, environmental monitoring, and precision medicine.

## 5 Conclusion

Odorant receptors, once considered specialized detectors of odorants in the nasal epithelium, are now recognized as a broadly expressed family of metabolite-sensitive GPCRs with functional roles across diverse human tissues. Rather than serving exclusively sensory functions, extra-nasal ORs expression reveals an unexpected and widespread chemosensory layer embedded within human physiology. The rapid accumulation of ORs structures and AI-based MD simulation, spanning both Class I and Class II receptors, now provides the near-atomic/atomic-level structure basis. These advances position extra-nasal OR as an emerging class of novel drug-candidates targeting GPCRs with broad implications for precise therapeutics. As structural, computational, and functional technologies continue to converge, ORs are poised to transition from understudied sensory proteins to central components of human disease biology and therapeutic innovation.
